# Dietary Betaine Impacts the Physiological Responses to Moderate Heat Conditions in a Dose Dependent Manner in Sheep

**DOI:** 10.3390/ani6090051

**Published:** 2016-08-29

**Authors:** Kristy DiGiacomo, Sarah Simpson, Brian J. Leury, Frank R. Dunshea

**Affiliations:** Faculty of Veterinary and Agricultural Sciences, The University of Melbourne, Parkville, VIC 3010, Australia; ssimpson88@gmail.com (S.S.); fdunshea@unimelb.edu.au (F.R.D.); brianjl@unimelb.edu.au (B.J.L.)

**Keywords:** betaine, heat stress, sheep, dose response, dietary supplement, temperature, physiology

## Abstract

**Simple Summary:**

Heat stress in sheep initiates physiological methods to dissipate heat that result in decreased production. This study investigated the use of a dietary supplement, the osmolyte betaine fed at two doses (2 or 4 g/day), on the physiological responses to heat in sheep. Heat exposure initiated physiological responses such as an increased rectal temperature and respiration rate as expected, while betaine supplemented at 2 g/day ameliorated these responses. Thus, dietary betaine supplementation may have beneficial effects for sheep exposed to heat.

**Abstract:**

Heat exposure (HE) results in decreased production in ruminant species and betaine is proposed as a dietary mitigation method. Merino ewes (*n* = 36, 40 kg, *n* = 6 per group) were maintained at thermoneutral (TN, *n* = 18, 21 °C) or cyclical HE (*n* = 18, 18–43 °C) conditions for 21 days, and supplemented with either 0 (control), 2 or 4 g betaine/day. Sheep had ad libitum access to water and were pair fed such that intake of sheep on the TN treatment matched that of HE animals. Heart rate (HR), respiration rate (RR), rectal (T_R_) and skin temperatures (T_S_) were measured 3 times daily (0900 h, 1300 h, 1700 h). Plasma samples were obtained on 8 days for glucose and NEFA analysis. The HE treatment increased T_R_ by 0.7 °C (40.1 vs. 39.4 °C for HE and TN respectively *p <* 0.001), T_S_ by +1.8 °C (39.3 vs. 37.5 °C, *p* < 0.001) and RR by +46 breaths/min (133 vs. 87 breaths/min, *p <* 0.001) compared to TN. The 2 g betaine/day treatment decreased T_R_ (39.8, 39.6 and 39.8 °C, *p <* 0.001), T_S_ (38.7, 38.0 and 38.5 °C, *p <* 0.001) and RR (114, 102 and 116 breaths/min for control, 2 and 4 g betaine/day, *p <* 0.001) compared to control. Betaine supplementation decreased plasma NEFA concentrations by ~25 μM (80, 55 and 54 μmol/L for 0, 2 and 4 g/day respectively, *p =* 0.05). These data indicate that dietary betaine supplementation at 2 g betaine/day provides improvements in physiological responses typical of ewes exposed to heat stress and may be a beneficial supplement for the management of sheep during summer.

## 1. Introduction

Temperature is one of the most important physiological stressors [1] and heat stress is a global problem for agriculture resulting in decreased reproduction, growth and production, increased health issues and increased mortality [2,3]. As environmental temperatures in Australia have been rising since the 1950s, particularly overnight; while the duration, intensity and frequency of extreme heat events have also been increasing (as reviewed by Alexander and Arblaster, 2009 [4]). It is generally expected that production animals in Australia will be exposed to more frequent chronic heat exposure (HE) in the future resulting in an increased need for effective mitigation strategies. Ruminants employ various methods to dissipate excess heat, including increased respiration rates (RR) and decreased activity and feed intake [5,6,7]. It is generally accepted that production animals in Australia are regularly exposed to both acute and chronic HE. As reviewed by Marai et al. (2007) [8], when experiencing thermoneutral temperatures sheep will lose approximately 20% of total body heat via respiration, and this will increase to 60% during heat stress. The basal RR and magnitude of increase in response to high ambient temperature is influenced by sheep breed whereby temperate breeds tend to have a greater basal RR compared to Middle Eastern breeds [8]. This can amount to an increased RR from the normal range of 20 to 40 breaths/min to 100 to 200 breaths/min when environmental temperatures exceed 35 °C [9]. This increase in activity of thermoregulatory mechanisms can therefore lead to increased water turnover and thus increased water requirements as demonstrated by Macfarlane*,* et al. (1958) [10] who noted a correlation between RR and water consumption per kg liveweight in Merino sheep.

Environmental modification is the simplest method for improving production during HE, yet the associated costs are high [5] and responses may not be beneficial in sheep [11]. Employing a dietary supplement may be a cost effective and simple method for ameliorating the negative impact of HE in sheep. Dietary betaine (trimethylglycine) is an amino acid capable of acting as an organic osmolyte or a methyl donor that can improve animal production measures in pigs [12,13,14,15,16], poultry [17,18], steers [19,20] and lambs [21]. This combination of effects suggests that betaine has the potential to ameliorate heat stress by reducing energy expenditure [22] and hence metabolic heat production, whilst also acting to maintain osmotic balance in animals experiencing HE. In pigs, the recommended dose of dietary betaine is 0.125% of intake although the effectiveness of greater doses for growth performance remains contentious [13,23]; while the effect of and dose responses to dietary betaine in sheep and other ruminants are poorly characterized. Rumen microbes consume betaine, although the reported volume and rate of the degradation of betaine is inconsistent between studies and likely influenced by base diet type (forage vs. grain) [24,25]. In lambs, dietary betaine supplemented at 2 g/kg feed had no effect on weight gain or final liveweight while subcutaneous fat thickness was decreased [21]. In a further study in lambs, betaine fed as either rumen escape betaine or feed grade betaine (both fed at 2 g/kg feed) did not alter final bodyweight or feed conversion rate compared to lambs fed control diets, while rumen escape betaine increased feed conversion rate when directly compared to lambs fed feed grade betaine [26]. In heifers, betaine supplemented as either lipid-coated betaine (fed at 4.2 g/d) or as concentrated separator by-product (fed at 15.5 g/d) produced no improvement to ADG; while over 60% of feed grade betaine remained after 24 h in in vitro observations of betaine degradation by rumen microbes fed a forage based diet [24]. Thus, there is variation in the doses of betaine fed in published experiments involving small and large ruminants and the optimal dose of supplemental dietary betaine for ruminants is yet to be elucidated. The aims of this study were to examine the effects of two doses of dietary betaine on physiological responses of sheep to controlled thermoneutral (TN) and HE conditions.

## 2. Materials and Methods

### 2.1. Animals and Treatments

All procedures used in this experiment were approved by The University of Melbourne’s School of Land and Environment Animal Ethics Committee (ID 1011620.2). Thirty-six 8 to 10-month-old Merino ewes (39.7 ± 3.1 kg; 2–3 cm fleece length) were selected from the same flock for this study. The experiment comprised three replicates in total with 6 sheep each for TN and HE environments (2 per betaine dose) and for an experimental period of 3 weeks. Animals were randomly assigned to one of the following doses of dietary betaine: 0 (control), 2 or 4 g betaine/day (Betaine 96% feed grade, FeedWorks, Romsey, Victoria, Australia). The betaine was supplemented daily at the morning feed prior to the feeding of the forage component of the diet to ensure that the full dose was consumed. The recommended doses of betaine in pigs is 0.125% of intake and in this experiment we selected increased doses (0.16 and 0.30% of feed intake respectively) to account for likely degradation by rumen microbes [25]. Sheep were acclimatized to the experimental facility for 14 days prior to the 3 week study and betaine was introduced into the diets at half the experimental concentration over the final 3 days of this period. Animals were fed a diet consisting of 70% alfalfa hay and 30% oat chaff (approximately 10 MJ of ME, CP 13%), split over twice daily feedings (0900 and 1600 h). As HE typically reduces feed intake, the TN sheep were pair-fed whereby intake was restricted to match ad libitum intakes of HE animals in an attempt to remove any confounding effects due to dissimilar nutrient intake [27,28,29]. Sheep exposed to HE were given ad libitum access to feed and their refusals were measured once daily by weighing orts. The amount (weight) of feed consumed by the HE pair member was then offered to their randomly selected TN partner on the following day and TN intake was also measured by weighing orts. Water was offered ad libitum and intake was measured twice daily (at feeding times) by measuring the volume consumed (in L) from the water bucket.

Animals were housed in metabolism crates within climate controlled rooms with the TN environment animals maintained at 21 (±0.7) °C and 57% ± 7.7% relative humidity (RH). The HE environment protocol began at 0900 h, after which the temperature increased (15 °C/h) to a maximum of 43 °C and 49% ± 11.8% RH where it remained until 1700 h; although due to fluctuations in the room temperature the average environmental temperature throughout the HE protocol (0900 to 1700 h) was 37 ± 3.5 °C. Overnight temperatures were returned to 21 ± 0.7 °C. The temperature humidity index (THI) was calculated using the formula

THI = db °C − [(0.31 − 0.31 RH)(db °C − 14.4)](1)

where db °C is the dry bulb temperature in °C and RH is the relative humidity percentage/100 as reported by Marai et al. (2007) [8].

### 2.2. Physiological Measures

Animals were weighed once per week prior to the feeding at 0900 h. Rectal (T_R_) and skin temperatures (T_S_), RR and heart rate (HR) were measured at 0900 h, 1300 h and 1700 h daily. Rectal temperatures were measured using a digital thermometer (Omron Australia MC34110, Port Melbourne, Victoria, Australia) inserted approximately 3 cm into the rectum and held in place until a stable temperature was obtained (approximately 30 s). The T_S_ was measured at the shoulder using a digital thermometer placed against the skin (by parting the wool) and held in place until a stable temperature was obtained. Respiration rates were measured by observing the number of flank movements in a 60 s period. Heart rates were measured using a stethoscope to count the number of beats in a 60 s interval.

### 2.3. Plasma Collection and Analysis

Blood samples were collected via jugular (anterior vena cava) venipuncture into a 10 mL lithium heparin Vacutainer^®^ (BD medical, North Ryde, New South Wales, Australia) at 1500 h (peak HE) on days 1, 3, 5, 8, 10, 12, 15 and 19 of the study. Samples were placed on ice and plasma collected after centrifugation at 3000 rpm for 10 min at 4 °C then frozen at −20 °C until subsequent analysis. Plasma glucose concentrations were measured using a glucose oxidase kit (Thermo Scientific, Scoresby, Victoria, Australia). Plasma NEFA concentrations were measured using a NEFA C kit (Wako, Novachem, Victoria, Australia) modified for use in 96 well plates [30]. Inter- and intra-assay coefficients of variation were 5.3% and 8.7%, and 4.7% and 7.4% for the glucose and NEFA assays, respectively.

### 2.4. Statistical Analysis

Growth performance data were analyzed for the effects of temperature, betaine supplementation amount and all interactions using the general ANOVA function in GenStat 15th edition [31]. The intake and weight gain responses were also analyzed for the pooled effect of betaine (0 g betaine/day vs. 2 + 4 g betaine/day) and within dose of betaine (2 vs. 4 g betaine/day) to elucidate if there was an overall effect of dietary betaine supplementation on growth performance. For the physiological measures, the data were analyzed by residual maximum likelihood (REML) function suitable for repeated measures with the fixed effects being dietary betaine, temperature, time and week and all appropriate interactions. Sheep and replicate were included as the random effects. For the plasma metabolites, the data were analyzed by REML function suitable for repeated measures with the fixed effects being dietary betaine, temperature and day, and sheep and replicate were included as the random effects. Appropriate interactions between the fixed effects were examined (betaine, temperature, time and day or week where appropriate). Results are reported as means separated by least significant differences and pooled standard error and means were considered to differ statistically when *p* ≤ 0.001 or *p* ≤ 0.05.

## 3. Results

### 3.1. Ambient Temperature, Intake and Weight

The THI for the TN environment was 20.1. For the HE environment the THI from 1700 h to 0900 h was 20.1 and from 0900 h to 1700 h was an average of 33.4 and a maximum of 38.5. There was no difference in feed intake between sheep maintained under TN or HE conditions (1242 vs. 1347 g/d for TN and HE, respectively; *p =* 0.060; Table 1). There was no effect (*p =* 0.66) of dietary betaine supplementation on feed intake (Table 1). Water consumption increased during HE (3.52 vs. 4.87 L/d for TN and HE, respectively; *p <* 0.001) but was unchanged (*p =* 0.20) by dietary betaine (Table 1). Daily gain was increased during HE (240 vs. 303 g/d for TN and HE, respectively; *p =* 0.016), possibly as a consequence of an increase in water intake as sheep were not fasted prior to weighing. While there was no significant effect of dietary betaine on daily gain (235 vs. 300 and 280 g/d for 0, 2 and 4 g betaine/day, respectively; *p =* 0.11), further analyses of the pooled betaine doses showed that dietary betaine increased daily gain by 23% (235 vs. 290 g/d for 0 and pooled (2 + 4) g betaine/day respectively; *p =* 0.040). There was no effect of either thermal environment (*p =* 0.17) or dietary betaine treatment (*p =* 0.18) on feed conversion efficiency (Table 1).

### 3.2. Physiological Responses to Temperature

**Figure 1:**
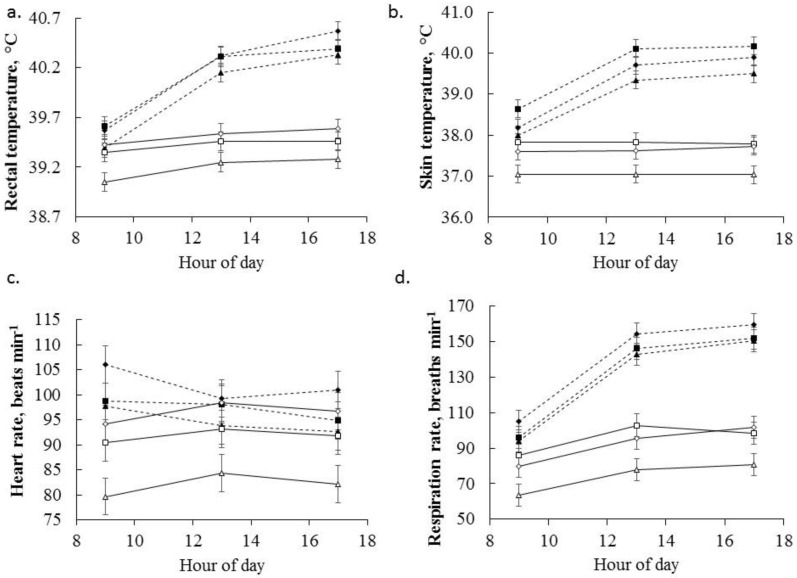
Mean (**a**) rectal temperature; (**b**) skin temperature; (**c**) heart rate and (**d**) respiration rate responses to 0 (□), 2 (Δ) and 4 (◊) g betaine/day in sheep (Merino ewe’s, *n* = 6 sheep per group) exposed to thermoneutral (TN) conditions (solid lines) and 0 (■), 2 (▲) and 4 (♦) g dietary betaine/day in sheep exposed to heat (HE) conditions (dashed lines). Data are mean ± sed for environmental temperature × betaine treatment × time of day pooled across the 21 d study. The *p*-values for the effects of time, environmental temperature and dietary betaine on rectal temperature and respiration rate were <0.001, <0.001 and <0.001 respectively. The *p*-values for the effects of time, environmental temperature and dietary betaine on skin temperature were <0.001, <0.001 and <0.001 respectively. The *p*-values for the effects of time, environmental temperature and dietary betaine on heart rate were <0.001, <0.001 and <0.001 respectively. See text for description of day effects and interactions.

The average T_R_ (across all time points and weeks) was lower in sheep exposed to the TN compared to HE environment (39.4 vs. 40.1 °C, sed 0.049, Figure 1a; *p <* 0.001). The T_R_ was greatest at 1700 h in HE sheep (39.3, 39.4 and 39.4 °C for TN and 39.5, 40.3 and 40.4 °C for HE at 0900 h, 1300 h and 1700 h respectively, sed 0.039, *p <* 0.001). Rectal temperature was influenced by experimental day (*p* < 0.001) and an interaction between day and temperature such that T_R_ was greater in HE sheep and increased with experimental day in both HE and TN sheep (data not shown). When summarized by week a similar pattern was noted such that T_R_ was greater in week 3 compared with weeks 1 and 2 (39.7, 39.7 and 39.8 °C for weeks 1 to 3 respectively, sed 0.065, *p* = 0.015). Over the course of the experiment, the 0900 h T_R_ did not change with experimental week (39.5 and 39.4 °C for weeks 1 vs. 3 respectively). Progressively, T_R_ increased at 1300 h and 1700 h as experimental week advanced (39.8 and 40.0 °C for weeks 1 and 3 at both 1300 and 1700 h respectively, sed 0.06, *p <* 0.001), indicating a general reduction in the ability of sheep to adapt to the chronic heat load.

The T_S_ was greater in sheep exposed to HE environment compared to TN sheep (37.5 vs. 39.3 °C*,* sed 0.112, *p* < 0.001), while T_S_ remained stable throughout the day in TN sheep but increased with time in response to HE (37.5, 37.5 and 37.5 °C for TN and 38.3, 39.7 and 39.9 °C for HE at 0900, 1300 and 1700 h respectively, sed 0.085, Figure 1b; *p <* 0.001). The T_S_ did not change due to experimental day (*p* = 0.31) and there were no significant interactions between experimental day and environmental temperature (*p* = 0.38). The T_S_ also did not differ by week (*p =* 0.28); however within each day T_S_ was greater at 1700 h compared to 0900 h and 1300 h (37.9, 38.6 and 38.7 °C for 0900, 1300 and 1700 h respectively, sed 0.028, *p* < 0.001).

Overall (as measured across all experimental weeks and times) sheep increased RR in response to HE compared with sheep exposed to TN conditions (87 vs. 133 breaths/min, Figure 1d; sed 3.19, *p <* 0.001). The RR remained stable throughout the afternoon for sheep in the TN treatment but was greater and increased throughout the day in sheep exposed to HE (76, 92 and 94 breaths/min for TN and 98, 148 and 154 breaths/min for 0900, 1300 and 1700 h respectively, sed 2.69, *p* < 0.001). The overall RR (irrespective of temperature) increased from experimental weeks 1 to 2 and remained stable for the final week of the experiment (105, 113 and 114 breaths/min for weeks 1–3 respectively, sed 1.44, *p* < 0.001) in line with the responses seen in T_R_; although there was no interaction between experimental week and temperature (*p* = 0.39). The RR differed due to experimental day (*p* < 0.001) such that the RR increased with experimental day, while there was no interaction between day and environmental temperature (*p* = 0.89).

The overall HR was increased in sheep exposed to HE conditions (90 vs. 98 beats/min, sed 1.90, *p* < 0.001). The HR of sheep exposed to TN conditions was lower throughout the day compared with HE sheep who had a consistently higher HR (88, 92 and 90 beats/min for TN and 101, 97 and 96 beats/min for 0900, 1300 and 1700 h respectively, Figure 1c; sed 1.63, *p <* 0.001). The HR fluctuated due to experimental day (*p* < 0.001, data not shown) and there was an interaction between day and environmental temperature (*p* = 0.045) such that HR was greater in HE sheep and fluctuated with experimental day but was lower on day 21 compared to day 1 for HE sheep but remained stable for TN sheep (data not shown). The HR did not change due to experimental week (*p* = 0.14).

### 3.3. Physiological Responses to Betaine Supplementation

There was no significant interaction between dietary betaine, temperature, time and day (*p* = 0.68) or dietary betaine, temperature and time on T_R_ (*p* = 0.24). Across the betaine treatments there was a strong effect of betaine dose (0 vs. 2 vs. 4 g betaine/day) on physiological parameters. Overall 2 g betaine/day reduced T_R_ compared with control and 4 g betaine/day treatments (39.8, 39.6 and 39.8 °C for 0, 2 and 4 g betaine/day respectively, sed 0.064, *p* < 0.001). Environmental temperature and betaine treatment did not interact to influence T_R_ (*p =* 0.75), although there was a significant relationship between betaine treatment and time such that 2 g betaine/day reduced T_R_ relative to both control and 4 g betaine/day treatments whereas 4 g betaine/day elevated T_R_ at 1700 h compared to control (39.5, 39.9 and 39.8 °C for control vs. 39.2, 39.7 and 39.8 °C for 2 g betaine/day vs. 39.5, 39.9 and 40.1 °C for 4 g betaine/day at 0900, 1300 and 1700 h respectively, Figure 1a; sed 0.056, *p <* 0.001). Additionally, there was a significant relationship between environmental temperature, betaine treatment and experimental week (*p* = 0.034) such that T_R_ (average across all measurement times) increased with experimental week for sheep exposed to TN conditions while this response was reduced in sheep fed 2 g betaine/day. Furthermore, this interaction showed that sheep fed 4 g betaine/day and exposed to TN conditions had a consistently greater T_R_ compared to those fed 0 or 2 g betaine/day; and while there was no difference in the T_R_ of sheep fed either 0 or 2 g betaine/day and exposed to HE, those fed 4 g betaine/day had a greater T_R_ compared with sheep fed both 0 and 2 g betaine/day (data not shown).

The effects of temperature, dietary betaine, time and day (*p* = 0.38) or temperature, dietary betaine and time did not interact to influence T_S_ (*p* = 0.77). The T_S_ was decreased in sheep receiving the betaine treatment (regardless of dose) compared with those fed the control diet, and this effect was greater in those fed 2 g compared to those fed 4 g betaine/day (38.7, 38.0 and 38.5 °C for 0, 2 and 4 g betaine/day respectively, sed 0.146, *p* < 0.001). While there was no interaction between environmental temperature and the betaine dose, there was a relationship between betaine dose and time such that T_S_ were consistently greater in sheep fed 0 g compared with those fed 2 g and 4 g betaine/day (38.2, 39.0 and 39.0 °C for control vs. 37.5, 38.2 and 38.3 °C for 2 g betaine/day vs. 37.9, 38.7 and 38.8 °C for 4 g betaine/day at 0900, 1300 and 1700 h respectively, sed 0.125, *p* = 0.025).

The effects of temperature, dietary betaine, time and day (*p* = 0.76) or temperature, dietary betaine and time (*p* = 0.22) did not interact to influence RR. Overall, sheep supplemented with 2 g betaine/day had a decreased RR compared to those fed 0 or 4 g betaine/day (114, 102 and 116 breaths/min for 0, 2 and 4 g betaine/day respectively; sed 4.09, *p <* 0.001). There was no interaction between dietary betaine treatment and environmental temperature on RR. While there was no interactive effects of temperature, dietary betaine, time and day (*p* = 0.096) or temperature, dietary betaine and time on HR (*p* = 0.23) there were dose dependent effects of betaine on pooled HR, with 2 and 4 g betaine/day being significantly lesser and greater respectively than control treatments (95, 88 and 99 beats/min for 0, 2 and 4 g betaine/day; sed 2.42, *p <* 0.001), while no interactions between betaine dose and time or environmental temperature were present.

### 3.4. Metabolite Responses

**Figure 2:**
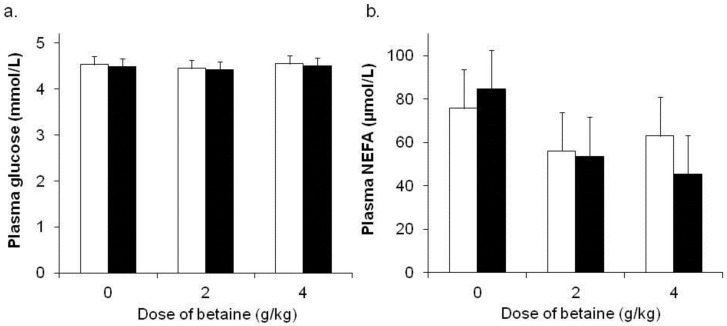
Plasma (**a**) glucose and (**b**) non-esterified fatty acid (NEFA) concentrations during thermoneutral (TN) (□) or heat (HE) (■) conditions in sheep (Merino ewe’s, *n* = 6 sheep per group) fed either 0, 2 or 4 g betaine/day for 21 days (data pooled across days for presentation). The *p*-values for the effects of environmental temperature and dietary betaine on plasma glucose were 0.49 and 0.95, respectively. The *p*-values for the effects of environmental temperature and dietary betaine on plasma NEFA were 0.66 and 0.05, respectively. The sed presented is for the interaction between temperature and dietary betaine. There were no significant 2- or 3-way interactions (*p >* 0.4). See text for description of day effects.

There was no effect of environmental temperature (*p =* 0.49) or dietary betaine (*p =* 0.95) on plasma glucose concentrations (Figure 2a). However, there was an effect of day on plasma glucose (*p =* 0.008) such that concentrations decreased with experimental day (data not shown). Plasma glucose concentrations were not influenced by an interaction between temperature, dietary betaine and sample day (*p* = 0.43). There was no effect of environmental temperature (*p =* 0.66) on plasma NEFA concentrations, whereas plasma NEFA were decreased by dietary betaine treatment (80, 55 and 54 μmol/L for 0, 2 and 4 g betaine/day, respectively; *p =* 0.05, Figure 2b). There was an effect of day on plasma NEFA such that plasma NEFA were greater (*p =* 0.004) on day 19 (the last day of sampling) than they were on previous days although there was no consistent pattern (i.e., there was not a consistent decrease across sample days) and plasma NEFA concentrations varied with sample day (data not shown). Plasma NEFA concentrations were not influenced by an interaction between temperature, dietary betaine and sample day (*p* = 0.52).

## 4. Discussion

These data clearly show that 2 g betaine/day can reduce the physiological measures of RR and T_R_ relative to controls under both TN and to a lesser degree HE conditions. However, when the dose was doubled to 4 g betaine/day there appeared to be an increased heat load in sheep as indicated by elevated T_R_, T_S_, RR and HR. Taken together, these responses demonstrate that betaine at the appropriate dose (2 g/day for these sheep) is a useful dietary additive to potentially reduce basal metabolic rates by decreasing energy loss via heat produced by processes such as respiration. Thus, betaine may be beneficial to ameliorate the effects of chronic HE, but quite clearly the response is dose dependent as greater doses can increase heat loads and potentially increase susceptibility to heat. Maintenance of a lower basal core temperature requires less energy and therefore 2 g betaine/day is likely to be energy sparing (although this was not directly measured in the present experiment). The use of nutritional interventions in the form of dietary supplements to mitigate the effects of heat stress in ruminants has been recently explored [32,33] and are suggested to be a useful mitigation tool, as supported by the findings presented here.

Increased RR is a common response to HE in sheep and this increase can account for up to 60% of heat loss [8]. Moreover, elevated T_R_ is commonly observed in heat stressed sheep [10] as a direct consequence of impaired thermoregulatory mechanisms, although when heat loss mechanisms are sufficient core (rectal) temperatures will remain stable. However, there is individual variation in the responses to heat (and solar radiation) in Merino sheep whereby sheep exposed to the sun did not have greater core body temperatures than those in the shade [11]. As the RR did not differ between shaded and unshaded sheep it is suggested that methods of evaporative cooling might not be observed by increases to RR and may involve increased evaporative cooling via a greater tidal and minute volumes [11]. The heat challenge imposed during this experiment was chosen as a reflection of moderate environmental temperatures that extensively housed sheep in Victoria (Australia) are expected to encounter in an average summer period. As reported by Marai et al. (2007) [8] a THI greater than 25.6 results in extreme to severe heat stress, meaning that the temperature challenge used in the HE treatment from 0900 to 1700 h in the present experiment was sufficient to initiate thermoregulatory responses (THI 33.4 to 38.5). However, by design, the cyclical pattern of the heat protocol utilized was cooler overnight (TN conditions) and thus allowed the sheep to offload excess heat overnight, although the physiological measurements remained increased in HE but not TN sheep at 0900 h indicating that the heat challenge imposed did not allow the sheep to sufficiently dissipate heat overnight. This explains the somewhat truncated rise in T_R_ measured, as supported by the findings of Collier*,* et al. (1981) [34] whereby dairy cows exposed to cooler overnight temperatures (<35 °C) were able to maintain a normal T_R_ compared to those exposed to a continual high heat. Nevertheless, the HE protocol initiated a physiological response as indicated by rises in T_R_, T_S_, RR and HR compared to TN conditions.

The results presented in this experiment support the findings of Alhidary et al. (2012) [35] in Merino wethers and Hopkins et al. (1978) [36] in Merino ewes where T_R_ increased in response to an increasing THI yet remained within the normal range (38.3 to 39.9 °C) for sheep [37]. Nevertheless, the significant increases in T_R_ in response to HE presented here were sufficient to suggest that the sheep experienced HS as discussed previously. Although the physiological responses during TN periods measured in the current experiment are greater than those described as normal in veterinary texts [37] where the normal resting RR for sheep (non-breed specific) is 16–34 breaths/min, the responses presented here (87 breaths/min) are supported by a study conducted by Chauhan*,* et al. (2014) [38] where a similar mean TN RR of 89.1 breaths/min was reported. However, these responses are greater than those reported by Sirkandakumar et al. (2003) [39] (50 breaths/min), Stockman et al. (2011) [40] (51 breaths/min) and Alhidary et al. (2012) [35] (40 breaths/min). This greater RR in TN treatment sheep is likely due to variations in sex, genetics and previous acclimation to environmental conditions. For example, as acclimation leads to reduced metabolic and heart rates and increased capacity of evaporative cooling systems [41] it is likely that sheep raised in warmer climates (such as those utilized in the studies by Stockman et al. (2011) [40] and Alhidary et al. (2012) [35] conducted in Western Australia and Queensland respectively) would have reduced resting (TN) RR and HR. However, while HE does not always result in clinical HS as indicated by a sustained rise in core temperature, it is likely that sub-clinical responses to HE are impacting upon the production efficiency of sheep. This was demonstrated by a previous experiment conducted by our research group whereby mild HE resulted in moderate increases to physiological measures (RR, T_R_ and T_S_) during HE in sheep but were associated with increased plasma prolactin concentrations and decreased insulin sensitivity [42].

Dietary betaine supplemented at 2 g betaine/day decreased RR in sheep compared to those fed 0 and 4 g betaine/day; and as T_R_ was also decreased in sheep fed 2 g betaine/day, it can be concluded that the sheep fed this treatment were either under a reduced heat load or were better able to dissipate heat compared to their counterparts fed 0 or 4 g betaine/day. Feed intake was not influenced by dietary betaine, indicating that reduced feed intake (and hence metabolic heat load due to the heat increment of feeding) did not contribute to the apparent reduction in heat load for sheep supplemented 2 g betaine/day. This response may in part be explained by the interactions between dietary betaine and water intake, although these responses were not significant in the present study. While there is limited information in ruminants, there is evidence of betaine influencing osmotic balance and energy expenditure in pigs. For example, porcine muscle tissue accumulates betaine when it is supplied in the diet, potentially indicating that muscle tissues would have either increased water retention or a reduction in the energy required to maintain osmotic gradients as a result [15]. As an organic osmolyte, betaine is known to improve water retention and reduce energy expenditure by reducing the need for cellular ion pumps and thus can be energy sparing [16,43,44]. Dietary betaine can improve lean tissue deposition in pigs [12], likely due to the increased energy available from the maintenance energy savings provided when betaine functions as an osmolyte. For example, as the gut incorporates betaine and is a large energy consumer, cellular betaine usage can potentially equate to whole body energy savings of ~8% [45]. In the present experiment daily gain was greater in animals fed betaine (combined effects of 2 and 4 g/day) compared to control animals, further suggesting that betaine is energy-sparing and able to reduce metabolic heat production and spare energy; although further studies are required to examine the effects of dietary betaine supplementation on production parameters in sheep.

Additionally, plasma NEFA concentrations decreased (~25 μM) in sheep supplemented with both doses of dietary betaine. This is consistent with observations from lactating dairy cows where plasma NEFA concentrations decreased with increasing dietary betaine supplementation amount [46]. Plasma NEFA concentrations were not influenced by HE in the present study, as supported by findings in Merino wethers [35] and lactating cattle [29,47]. Thus, the reduction in plasma NEFA is not the result of the thermal conditions utilized and is likely the result of betaine inhibiting lipogenesis or increasing tissue NEFA clearance rates. Betaine can enhance the synthesis of methylated compounds such as phospholipids and therefore can directly influence lipid metabolism [48]. According to Wray-Cahen et al. (2004) [23] and Huang et al. (2008) [48], dietary betaine supplementation in pigs influences lipid metabolism via reducing adipose accretion rather than influencing oxidation. Huang et al. (2008) [48] demonstrated that dietary betaine supplementation reduces the activity of key enzymes (such as fatty acid synthase, FAS) that regulate fatty acid accretion in porcine subcutaneous adipose tissue. This is supported by responses in steers fed either 10 or 20 g of betaine in which a reduction in FAS activity in subcutaneous adipose tissue [20] was observed. Conversely, FAS activity has also been shown to increase in steers fed 40 g betaine [20]. Therefore, the effects of dietary betaine supplementation on fatty acids may occur in a dose dependent manner, as also noted in lactating dairy cattle as described by Dunshea et al. (2013) [32]. Additionally, dietary betaine has been shown to influence hepatic function by modifying insulin signaling pathways and adipose tissue function by inhibiting lipogenesis [49,50,51,52]. As HE reduces hepatic blood flow due to the redistribution of blood towards the extremities to aid in heat dissipation the 2 g betaine/day may be ameliorating this effect. However, hepatic fuel oxidation varies considerably between ruminant and non-ruminant species meaning it is likely that the effects of betaine will vary in sheep and cattle compared to those measured in pigs. Conversely, in the present study there was a reduction in T_S_ in sheep fed both 2 and 4 g betaine/day compared to control, indicating that betaine may influence blood flow to the periphery. This could be due to an increase in hepatic blood flow as suggested, although rumen protected betaine did not alter portal or hepatic venous blood flow in goat weathers [53]. Further research into the metabolic effects of betaine in ruminants, particularly in adipose and hepatic tissues, is warranted.

As sheep supplemented with 4 g betaine/day had overall (regardless of environmental temperature) increased T_R_ at 1700 h and a greater overall weekly T_R_ under both TN and HE conditions (Figure 1) compared to the other treatments it can be concluded that these sheep were either under an increased metabolic heat load or had a reduced capacity to dissipate heat. Therefore, the 4 g betaine/day appears to be beyond the dose response curve and is no longer eliciting beneficial effects. This is likely due to an insufficient initiation of physiological heat dissipation methods as demonstrated by a lack of response measured by RR. Thus, the insufficient heat loss mechanisms contribute a heat load on the animal and in part explain the increased core temperature in both HE and TN sheep supplemented with 4 g betaine/day. Somewhat similar responses have been noted in lactating dairy cattle where betaine successfully improved milk yield during TN conditions, while cows exposed to HE and supplemented with betaine (35 g/day) had lower RR but greater vaginal temperatures [32]. These combined results are perhaps suggesting that high doses of dietary betaine increase metabolic heat production that contributes to the already increased heat load on ruminants exposed to heat.

## 5. Conclusions

The data presented in this experiment demonstrated the novel finding that there are dose dependent responses to dietary betaine supplementation in sheep. At the appropriate concentration, dietary betaine may assist sheep to counter HE as dietary betaine supplemented at 2 g betaine/day (0.16% of feed intake) decreased physiological measures. However, at the greater 4 g betaine/day concentration T_R_ was increased, suggesting that there are metabolic responses to dietary betaine that are yet to be elucidated. Further examination of the effects of different does of betaine in sheep, with particular focus on production and metabolism, are warranted. Additionally, as this experiment has suggested an appropriate supplementation dose for dietary betaine further experiments examining the responses to 2 g betaine/day in larger field studies in both HE (summer) and TN (winter) conditions is justified to explore the production potential of this supplement. Finally, further examination of the responses to betaine in dairy and beef animals is also warranted as these species are also susceptible to HE associated production losses.

## Figures and Tables

**Table 1:** Effect of temperature (*T*) and dietary betaine (B) supplementation on growth performance of sheep (Merino ewe’s, *n* = 6 sheep per group) over the 21 day treatment period.

Temperature (*T*)	Thermoneutral (TN)			Heat (HE)				*p*-Value ^1^			
Betaine (B), g/d	0	2	4	0	2	4	Sed ^a^	T	B	0 vs. 2 + 4	2 vs. 4
Feed intake, g/d	1171	1237	1317	1371	1329	1342	94.1	0.060	0.66	0.56	0.51
Water intake, L/d	3.21	3.38	3.97	4.84	4.61	5.16	0.504	<0.001	0.20	0.40	0.11
Daily gain, g/d	194	271	261	279	331	305	43.7	0.016	0.11	0.04	0.55
Feed conversion efficiency	0.17	0.22	0.20	0.20	0.25	0.20	0.038	0.15	0.18	0.10	0.38

^a^ Standard error of the difference for T × B group; ^1^ There were no significant (*p* > 0.43) T × B interactions.

## References

[1] Horowitz M. (2002). From molecular and cellular to integrative heat defense during exposure to chronic heat. Comp. Biochem. Physiol. A Comp. Physiol..

[2] St-Pierre N.R., Cobanov B., Schnitkey G. (2003). Economic losses from heat stress by US livestock industries. J. Dairy Sci..

[3] Vitali A., Segnalini M., Bertocchi L., Bernabucci U., Nardone A., Lacetera N. (2009). Seasonal pattern of mortality and relationships between mortality and temperature-humidity index in dairy cows. J. Dairy Sci..

[4] Alexander L.V., Arblaster J.M. (2009). Assessing trends in observed and modelled climate extremes over Australia in relation to future projections. Int. J. Climatol..

[5] Collier R.J., Zimbelman R.B. (2007). Heat stress effects on cattle: What we know and what we don‘t know. Proceedings of the 22nd Annual Southwest Nutrition & Management Conference.

[6] Hahn G.L. (1999). Dynamic responses of cattle to thermal heat loads. J. Anim. Sci..

[7] Mader T.L. (2003). Environmental stress in confined beef cattle. J. Anim. Sci..

[8] Marai I.F.M., El-Darawany A.A., Fadiel A., Abdel-Hafez M.A.M. (2007). Physiological traits as affected by heat stress in sheep—A review. Small Rumin. Res..

[9] Hales J.R.S., Brown G.D. (1974). Net energetic and thermoregulatory efficiency during panting in the sheep. Comp. Biochem. Physiol. A Comp. Physiol..

[10] Macfarlane W.V., Morris R.J.H., Howard B. (1958). Heat and water in tropical Merino sheep. Aust. J. Agric. Res..

[11] Johnson K.G. (1991). Body temperatures and respiratory rates of free-ranging Merino sheep in and out of shade during summer. Aust. J. Agric. Res..

[12] Dunshea F.R., Cadogan D.J., Partridge G.G. (2009). Dietary betaine and ractopamine combine to increase lean tissue deposition in finisher pigs, particularly gilts. Anim. Prod. Sci..

[13] Fernandez-Figares I., Wray-Cahen D., Steele N.C., Campbell R.G., Hall D.D., Virtanen E., Caperna T.J. (2002). Effect of dietary betaine on nutrient utilization and partitioning in the young growing feed restricted pig. J. Anim. Sci..

[14] Lawrence B.V., Schinckel A.P., Adeola O., Cera K. (2002). Impact of betaine on pig finishing performance and carcass composition. J. Anim. Sci..

[15] Matthews J.O., Southern L.L., Higbie A.D., Persica M.A., Bidner T.D. (2001). Effects of betaine on growth, carcass characteristics, pork quality and plasma metabolites of finishing pigs. J. Anim. Sci..

[16] Suster D., Leury B.J., King R.H., Mottram M., Dunshea F.R. (2004). Interrelationships between porcine somatotropin (pST), betaine, and energy level on body composition and tissue distribution of finisher boars. Aust. J. Agric. Res..

[17] Wang Y.Z., Xu Z.R., Feng J. (2004). The effect of betaine and DL-methionine on growth performance and carcass characteristics in meat ducks. Anim. Feed Sci. Technol..

[18] Zhan X.A., Li J.X., Xu Z.R., Zhao R.Q. (2006). Effects of methionine and betaine supplementation on growth performance, carcase composition and metabolism of lipids in male broilers. Br. Poult. Sci..

[19] Bock B.J., Brethour J.R., Harmoney K.R., Goodall S.R. (2004). Influence of betaine on pasture, finishing, and carcass performance in steers. Prof. Anim. Sci..

[20] DiGiacomo K., Warner R.D., Leury B.J., Gaughan J.B., Dunshea F.R. (2014). Dietary betaine supplementation has energy-sparing effects in feedlot cattle during summer, particularly in those without access to shade. Anim. Prod. Sci..

[21] Fernandez C., Gallego L., Lopez-Bote C.J. (1998). Effect of betaine on fat content in growing lambs. Anim. Feed Sci. Technol..

[22] Moeckel G.W., Shadman R., Fogel J.M., Sadrzadeh S.M.H. (2002). Organic osmolytes betaine, sorbitol and inositol are potent inhibitors of erythrocyte membrane ATPases. Life Sci..

[23] Wray-Cahen D., Fernández-Fígares I., Virtanen E., Steele N.C., Caperna T.J. (2004). Betaine improves growth, but does not induce whole body or hepatic palmitate oxidation in swine (Sus scrofa domestica). Comp. Biochem. Physiol. A Comp. Physiol..

[24] Loest C.A., Titgemeyer E.C., Drouillard J.S., Blasi D.A., Bindel D.J. (2001). Soybean hulls as a primary ingredient in forage-free diets for limit-fed growing cattle. J. Anim. Sci..

[25] Mitchell A.D., Chappell A., Knox K.L. (1979). Metabolism of betaine in the ruminant. J. Anim. Sci..

[26] Fernández C., López-Saez A., Gallego L., de la Fuente J.M. (2000). Effect of source of betaine on growth performance and carcass traits in lambs. Anim. Feed Sci. Technol..

[27] Baumgard L.H., Wheelock J.B., O‘Brien M., Schwartz G., Zimbelman R.B., Sanders S.R., VanBaale M.J., Collier R.J., Rhoads M.L., Rhoads R.P. The differential effects of heat stress vs. underfeeding on production and post-absorptive nutrient partitioning. Proceedings of the 22nd Annual Southwest Nutrition & Management Conference.

[28] Rhoads M.L., Kim J.W., Collier R.J., Crooker B.A., Boisclair Y.R., Baumgard L.H., Rhoads R.P. (2009). Effects of heat stress and nutrition on lactating Holstein cows: II. Aspects of hepatic growth hormone responsiveness. J. Dairy Sci..

[29] Rhoads M.L., Rhoads R.P., VanBaale M.J., Collier R.J., Sanders S.R., Weber W.J., Crooker B.A., Baumgard L.H. (2009). Effects of heat stress and plane of nutrition on lactating Holstein cows: I. Production, metabolism, and aspects of circulating somatotropin. J. Dairy Sci..

[30] Johnson M.M., Peters J.P. (1993). Technical note: An improved method to quantify nonesterified fatty acids in bovine plasma. J. Anim. Sci..

[31] Payne R.W., Harding S.A., Murray D.A., Soutar D.M., Baird D.B., Welham S.J., Kane A.F., Gilmour R., Thompson R., Webster R. (2008). GenStat Release 11 Reference Manual.

[32] Dunshea F.R., Leury B.J., Fahri F., DiGiacomo K., Hung A., Chauhan S., Clarke I.J., Collier R., Little S., Baumgard L.H. (2013). Amelioration of thermal stress impacts in dairy cows. Anim. Prod. Sci..

[33] Rhoads R.P., Baumgard L.H., Suagee J.K., Sanders S.R. (2013). Nutritional interventions to alleviate the negative consequences of heat stress. Adv. Nutr..

[34] Collier R.J., Eley R.M., Sharma A.K., Pereira R.M., Buffington D.E. (1981). Shade management in subtropical environment for milk yield and composition in Holstein and Jersey cows. J. Dairy Sci..

[35] Alhidary I.A., Shini S., Al Jassim R.A.M., Gaughan J.B. (2012). Physiological responses of Australian Merino wethers exposed to high heat load. J. Anim. Sci..

[36] Hopkins P., Knights G., Feuvre A.L. (1978). Studies of the environmental physiology of tropical Merinos. Aust. J. Agric. Res..

[37] Kahn C.M., Line S. (2010). The Merck Veterinary Manual.

[38] Chauhan S.S., Celi P., Leury B.J., Clarke I.J., Dunshea F.R. (2014). Dietary antioxidants at supranutritional doses improve oxidative status and reduce the negative effects of heat stress in sheep. J. Anim. Sci..

[39] Srikandakumar A., Johnson E.H., Mahgoub O. (2003). Effect of heat stress on respiratory rate, rectal temperature and blood chemistry in Omani and Australian Merino sheep. Small Rumin. Res..

[40] Stockman C.A., Barnes A.L., Maloney S.K., Taylor E., McCarthy M., Pethick D. (2011). Effect of prolonged exposure to continuous heat and humidity similar to long haul live export voyages in Merino wethers. Anim. Prod. Sci..

[41] Horowitz M. (2001). Heat acclimation: Phenotypic plasticity and cues to the underlying molecular mechanisms. J. Therm. Biol..

[42] DiGiacomo K., Dunshea F.R., Leury B.J. (2012). Mild heat load alters some aspects of nutrient partitioning and gene expression in sheep. Proceedings of the 63rd Annual Meeting of the European Federation of Animal Science.

[43] Caldas T., Demont-Caulet N., Ghazi A., Richarme G. (1999). Thermoprotection by glycine betaine and choline. Microbiology.

[44] Craig S.A.S. (2004). Betaine in human nutrition. Am. J. Clin. Nutr..

[45] Cronje P. (2005). Heat stress in livestock—The role of the gut in its aetiology and a potential role for betaine in its alleviation. Recent Adv. Anim. Nutr. Aust..

[46] Wang C., Liu Q., Yang W.Z., Wu J., Zhang W.W., Zhang P., Dong K.H., Huang Y.X. (2010). Effects of betaine supplementation on rumen fermentation, lactation performance, feed digestibilities and plasma characteristics in dairy cows. J. Agric. Sci..

[47] Shwartz G., Rhoads M.L., VanBaale M.J., Rhoads R.P., Baumgard L.H. (2009). Effects of a supplemental yeast culture on heat-stressed lactating Holstein cows. J. Dairy Sci..

[48] Huang Q.C., Xu Z.R., Han X.Y., Li W.F. (2008). Effect of dietary betaine supplementation on lipogenic enzyme activities and fatty acid synthase mRNA expression in finishing pigs. Anim. Feed Sci. Technol..

[49] Abdelmalek M.F., Sanderson S.O., Angulo P., Soldevila-Pico C., Liu C., Peter J., Keach J., Cave M., Chen T., McClain C.J. (2009). Betaine for nonalcoholic fatty liver disease: Results of a randomized placebo-controlled trial. Hepatology.

[50] Borgschulte G., Kathirvel E., Herrera M., French S.W., Morgan T.R., Morgan K., Bottiglieri T. (2008). M1769 Betaine treatment reverses insulin resistance and fatty liver disease without reducing oxidative stress or endoplasmic reticulum stress in an animal model of NAFLD. Gastroenterology.

[51] Ji C., Kaplowitz N. (2003). Betaine decreases hyperhomocysteinemia, endoplasmic reticulum stress, and liver injury in alcohol-fed mice. Gastroenterology.

[52] Wang Z., Yao T., Pini M., Zhou Z., Fantuzzi G., Song Z. (2010). Betaine improved adipose tissue function in mice fed a high fat diet: A mechanism for hepatoprotective effect of betaine in nonalcholic fatty liver disease. Am. J. Physiol. Gastrointest. Liver Physiol..

[53] Banskalieva V., Puchala R., Goetsch A.L., Luo J., Sahlu T. (2005). Effects of ruminally protected betaine and choline on net flux of nutrients across the portal-drained viscera and liver of meat goat wethers consuming diets differing in protein concentration. Small Rumin. Res..

